# An Economic Approach to Animal Models of Alcoholism

**Published:** 2000

**Authors:** Gene M. Heyman

**Affiliations:** Gene M. Heyman, Ph.D., is a lecturer in the Department of Psychiatry, Harvard Medical School, Cambridge, and a research psychologist in the Behavioral Psychopharmacology Research Laboratory, East House 3, McLean Hospital, Belmont, Massachusetts

**Keywords:** animal model, economic theory of AODU (AOD [alcohol or other drug] use, abuse, and dependence), economic elasticity, AOD price, AOD use pattern, AOD preference, taste conditioning, operant conditioning, choice-making behavior

## Abstract

Researchers have long sought an animal model for human alcohol consumption. This article describes an economic-based approach to a model of alcohol preference in rats. The procedures are based on an analogy between clinical accounts of human drinking and the economic analysis of consumption. Both clinical and economic investigators typically define consumption patterns in terms of the influence of negative consequences. For example, the clinical account emphasizes the persistence of heavy drinking despite mounting alcohol-related aversive consequences, and in economic analyses, the term “inelastic demand” is used to refer to the persistence of consumption despite large increases in prices. In the experimental procedure described here, rats worked for alcohol and food. Presses on one lever earned a drink of 10 percent alcohol plus saccharin, and presses on a second lever earned isocaloric drinks of a starch solution. After behavior stabilized, the response requirements (which are analogous to prices) for one or both drinks were increased. The rats maintained baseline alcohol consumption levels despite large increases in the “price” of alcohol. In contrast, the same price increases markedly reduced starch intake. That is, food consumption was sensitive to price hikes, but alcohol consumption was not. The results demonstrate that a common economic framework can be used to describe human and animal behavior and, hence, the possibility of an animal model of human alcohol consumption. The article also points out that economic concepts provide a framework for understanding a wide range of human drinking patterns, including controlled social drinking and excessive alcoholic drinking.

For more than half a century, researchers have been plying rats with alcohol in the hope of developing a valid animal model of human alcohol consumption (e.g., Richter and Campbell 1940). Following up on the observation that alcoholism tends to run in families, one strategy has been to breed alcohol-consuming rats (e.g., Li and Lumeng 1984). Other research facilities have focused on drinking history. For example, Samson and his colleagues (1988) found that rats that drank sweetened alcoholic drinks would subsequently drink larger amounts of unsweetened alcohol solutions. Our approach has been to manipulate the economic conditions governing access to an alcoholic drink. The rats were placed in a setting in which lever presses would earn either a sweetened alcohol drink or food. We then varied the number of times the rats had to press the levers in order to obtain alcohol, food, or both. In this way, it was possible to examine the relationship between the “price,” defined as the lever press requirement, and alcohol consumption. For example, would an increase in price have more of an effect on alcohol consumption or on food consumption? The theoretical background for this approach includes clinical accounts of drinking, such as those provided by the American Psychiatric Association (APA)(1994), and elementary economic ideas concerning the relationship between changes in price and changes in consumption. This article begins with the clinical account of alcohol consumption, as it sets the stage for this and all animal models of human drinking.

## Clinical Account of Drinking and the Economic Account of Consumption: An Analogy

The APA publishes a widely used diagnostic manual (i.e., the *Diagnostic and Statistical Manual of Mental Disorders* or DSM [1994]) that includes a set of criteria for identifying individuals as “abusers” of alcohol or as “dependent” on alcohol. The diagnoses are based on expert opinion and field research, and for many syndromes, including problem drinking, they yield a substantial degree of inter-rater reliability (e.g., Spitzer et al. 1978). For instance, there is more than an 80-percent likelihood that different clinicians will agree whether a particular individual meets or does not meet the criteria for “alcohol dependence” (e.g., Helzer et al. 1977; Robins et al. 1982). Thus, the manual’s account of human drinking problems provides a reasonable target for animal procedures.

According to the most recent edition of the DSM (APA 1994), problem drinking is defined primarily by the degree to which drinking persists in the face of alcohol-related aversive events. Those who abuse alcohol or are dependent on alcohol repeatedly get drunk despite previous drinking-related problems, such as arrests, fights, and poor job performance (p. 196). In addition, the alcohol dependent drinker, who is considered to have a more serious problem, develops tolerance to alcohol’s intoxicating effects, suffers withdrawal symptoms and, when attempting to quit drinking, relapses back to heavy alcohol use. (In other words, “alcohol dependence” approximates the more commonly used term “alcoholism.”)

The influence of alcohol’s negative consequences is also used to identify social or nonproblem drinkers. The DSM states it this way (p. 194):

as many as 90% of adults in the United States had some experience with alcohol, and a substantial number (60% of males and 30% of females) have had one or more alcohol-related adverse events (e.g., driving after consuming too much alcohol, missing school or work due to a hangover). Fortunately, most individuals learn from these experiences to moderate their drinking and do not develop Alcohol Dependence or Abuse.

Thus, the influence of drinking-related problems on future drinking is the criterion for differentiating healthy and unhealthy drinking patterns, with the healthy taking due heed.

Although the purpose of the DSM account is to help clinicians and researchers differentiate problem and non-problem drinkers, the logic of the approach follows closely the logic of the economic analysis of consumption. The key terms in the economic approach are changes in price and changes in the level of consumption. For many goods, a change in price has a profound effect on consumption. For example, studies show that when the price of green peas or restaurant meals increases, consumption of these commodities decreases, and the relative size of the change in consumption is considerably larger than the relative size of the change in price (Houthakker and Taylor 1970; Frank 1991). The technical term for these examples is “elastic demand,” with “elastic” referring to the fact that consumption ranges widely in response to relatively small increments or decrements in price.

However, consumption is not always sensitive to changes in price. For example, when the price of health care or gasoline goes up by say 100 percent, the decreases in consumption are typically less than 50 percent (e.g., Nicholson 1985). As a result, the consumer spends more on the goods but gets less. The technical term for this situation is “inelastic demand.” Although the terms (“elastic” and “inelastic”) suggest a continuum, there is a qualitative difference. Instances of “inelastic demand” are at best unpleasant and when persistent or extreme inspire action to “correct” the markets, as in the call for government regulation of health costs or the recent blockade of fuel depots by French truck drivers protesting soaring gasoline prices. This is relevant because there is a connection between inelastic demand and problem drinking.

### Inelastic Demand for Alcohol as a Component of Problem Drinking

The logic of elasticity of demand parallels the logic of the DSM’s description of drinking patterns. Both accounts focus on the relationship between behavioral consequences and the future likelihood of the behavior. When the DSM authors state that a social drinker is someone who is influenced by the aversive consequences of alcohol, they are in effect saying that for social drinkers, demand for alcohol is relatively elastic. For instance, by definition, social drinking occurs under socially approved conditions, and it ceases to be social drinking if consumption repeatedly leads to extreme intoxication or debilitating hangovers. Similarly, to say that those who are alcohol abusers or alcohol dependent continue to drink heavily despite past alcohol-dependent aversive consequences is to say that alcohol consumption is inelastic. It persists, despite increased and mounting costs. The analogy does not, of course, cover all of human drinking. For instance, it treats the drinker (and the rat) as a “black box,” omitting the biological and psychological correlates of drinking. However, this is the proper starting point for an animal model. That is, before exploring the biology and psychology of rat drinking, there should first be some basis for expecting that the mediating mechanisms will be relevant to the human case.

### Methodological Issues: Calories and Taste

Alcohol is a rich source of calories and has a unique taste. These factors cannot be removed, so the experimenter must somehow minimize or neutralize their influence to establish that alcohol’s pharmacological effects are influencing behavior. To reduce the influence of calories, we arranged a concurrent highly palatable food that provided the same number of calories as the alcohol. For instance, in all of the studies discussed in this report, the rats had access to solutions of starch or sucrose as well as alcohol. The point was to see if changes in the experimental conditions brought about similar or different changes in alcohol and food consumption. If the consumption patterns proved similar, then the underlying motivational basis must be similar. If the consumption patterns proved to be different, then it is reasonable to infer that the mechanisms governing consumption differed. For example, increases in body weight decrease food consumption. Would they also decrease alcohol consumption? Alternatively, “free” alcohol decreases alcohol-motivated behavior. Would it also decrease food-motivated behavior?

To reduce the influence of alcohol’s aversive taste and to make alcohol taste more like the control solutions, we mixed it with saccharin. As most researchers working with animal models provide their subjects alcohol mixed with water, this aspect of the procedure deserves some additional background.

Alcohol mixed with water stimulates both “sweet” and “bitter” reactions (Kiefer et al. 1990), and as concentration increases, the bitter reaction dominates (e.g., Richter and Campbell 1940). However, despite these facts, the tradition is to present rats alcohol mixed with water. The following facts persuaded us that palatability was the better course.

The addition of saccharin more than doubled alcohol intake, producing blood alcohol levels that were two to four times greater than obtained with selectively bred, alcohol preferring (P) rats under otherwise similar circumstance (compare Heyman et al. 1996 with Schwarz-Stevens et al. 1991). As pharmacological influence on consumption is a function of blood alcohol levels, this difference means that the saccharin procedure is more likely to lead to pharmacologically based preferences. In support of this point, it has not proved possible to motivate rats to work for alcohol mixed with water when there is a palatable food like sugar available. Moreover, this is the case even for selectively bred, alcohol preferring (or P) rats (e.g., Schwarz-Stevens et al. 1991). In contrast, commercially bred as well as selectively bred alcohol-preferring (P) and alcohol-nonpreferring (NP) rats readily drink large amounts of alcohol plus saccharin in the presence of palatable and preferred foods (e.g., Heyman 2000). These findings provide the researcher with a critical methodological advantage. If the rats continue to drink alcohol and saccharin mixtures when they also have access to palatable foods, then it is possible to introduce control solutions that provide the same caloric density as alcohol. Thus, we were able to test if alcohol consumption was motivated by calories or by its pharmacology.

A corollary of this discussion is that the researcher does not remove the influence of taste by mixing alcohol with water. Rather, the researcher has the option of providing his or her subjects with a more or less aversive-tasting alcohol solution. The evidence to date supports the more palatable approach.

## The Experiments: Response Requirement as Price

We conducted a series of experiments in which the price of sweetened alcohol drinks and the price of sucrose and starch solutions were varied. In some studies, the price of each substance was changed one at a time. In others, the price of each commodity was changed in tandem. The “tandem” study will be given the most attention as it reveals the behavioral processes most clearly.

The experimental chamber held two levers and two dippers. At one lever the rats could earn a drink of 10 percent alcohol plus saccharin. At the other lever the rats could earn a drink of starch solution (i.e., Polycose), with the concentration set to provide the same number of calories per milliliter (mL) as the alcohol (“isocaloric solutions”). Price was represented by the response requirement for earning a drink. In the initial condition of the study, the requirement was 5 responses for both the alcohol solution and the starch solution. For instance, in a 45-minute session, there were about 600 alcohol lever presses and about 1,200 starch lever presses so that the rats earned approximately 12 mL of 10 percent alcohol solution and 24 mL of starch solution. This is a very high rate of intake. For instance, it is equivalent (by weight) to a 165-pound person drinking about 6.4 ounces of pure alcohol, or two six packs of domestic beer, in 45 minutes. (Each mL of alcohol provides about 0.79 grams of alcohol, there are about 28.6 grams to an ounce, the rats weighed about 390 grams, and the alcohol content of beer varies between about 4 and 5 percent.) In subsequent conditions, the prices were increased in 50 or 100 percent steps, leading to a final set of prices of 30 lever presses for each drink. Each price was in effect until consumption levels stabilized.

### Price and Income

The nominal experimental manipulation was a change in response requirements. If this is seen as a change in price then there is the additional implication of a change in real income. For instance, under ordinary circumstances increases in prices and a reduction in the monthly paycheck are equivalent in that both reduce purchasing power. But what is the equivalent of “income” for rats that earn their keep by pressing levers? The closest analogy is the number of possible alcohol and food drinks. For instance, since the session length was fixed at 45 minutes, increases in the response requirements invariably decreased the total number of possible starch and alcohol servings. Thus, changes in consumption patterns may reflect changes in the overall availability of alcohol and food as well as changes in price. (Note that price and commodity availability [income] are logically distinct. For example, we could have increased price and kept the overall availability of food and alcohol constant by increasing session length in proportion to the price increases.)

### Changes in Price Had Less Influence on Alcohol Consumption

**Figure 1:**
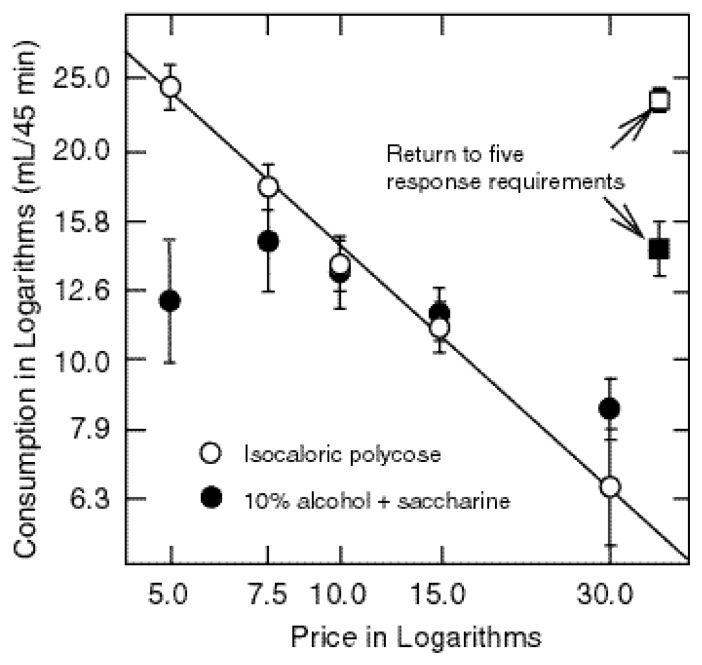
Increases in alcohol “price” decreased the amount of starch that rats consumed but failed to decrease their alcohol consumption except at the highest alcohol price level (i.e., 30 responses). The data are graphed in logarithmic coordinates to show that a linear plot occurred when relative change in consumption was proportional to relative price increase. This finding, however, was true for starch consumption but not for alcohol consumption. As indicated by the data points, approximately seven rats dominated the last three sessions of each condition.

The relationship between increases in the response requirements and changes in alcohol and food consumption is shown in figure 1 below. The axes are logarithmic because in econometric studies this transformation often yields a linear relationship between the variables. As was expected, price increases decreased food consumption. Each doubling of the price reduced starch consumption by about 40 percent of its current value. The relationship was linear (in logarithmic coordinates), meaning that relative increases in price had about the same relative effect on food consumption over the entire range of prices. Also note that the relative decrease in food intake was somewhat less than the relative increase in price so that demand for food was somewhat inelastic. (The slope was less than −1.0.)

**Figure 2:**
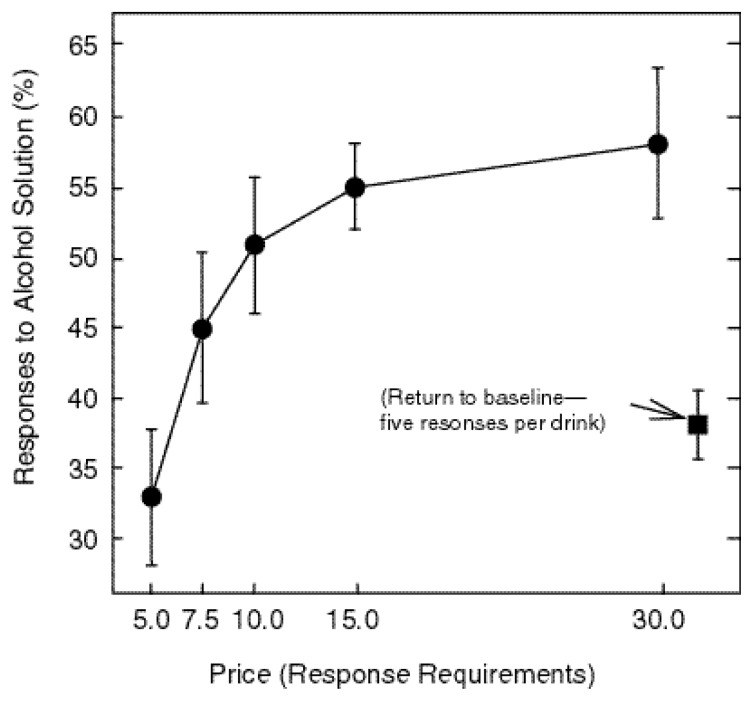
Preference for the alcohol solution as a function of “price” change among study rats. The x-axis displays the response requirement and the y-axis displays the proportion of responses at the alcohol lever. The data points show the average response of seven rats during the final three sessions of each condition.

Price increases had a quite different effect on alcohol consumption. For the first four price hikes, alcohol consumption either increased or stayed about the same. Indeed, it was not until the response requirement had increased by 500 percent relative to baseline, 30 responses per drink, that alcohol intake declined. As alcohol consumption both increased and decreased, a straight line does not describe the results. However, for purposes of comparison, a line was fit to the data points. The slope did not differ significantly from 0.0. In other words, increases in price did not systematically decrease alcohol consumption, which is to say that demand for the alcohol solution was highly inelastic. This implies that there must have been marked increases in responding at the alcohol lever (see figure 2).

Figure 2 shows lever pressing for alcohol and for food. Again the response requirement (i.e., “price”) is plotted on the x-axis, but now the proportion of responses allocated to the lever that operated the alcohol dipper is plotted on the y-axis. When each substance cost just five responses, the alcohol lever was chosen about 33 percent of the time. However, as price increased, responding shifted to alcohol, so that the overall response allocation ended up in favor of the alcohol solution. Thus, there was a powerful correlation between increases in price, decreases in income, and increases in alcohol seeking.

These results are representative of a series of experiments in which prices and income were varied (e.g., Petry and Heyman 1995). In each study, manipulations that produced substantial decreases in food consumption had little influence on alcohol consumption. That is, demand for alcohol was always greater than was demand for food, and in each study, preference shifted from food to alcohol as prices increased or income decreased. Importantly, the results did not have to work out this way. Alcohol consumption could have decreased as much or more than did food intake. For example, in a similar experiment in which lever presses produced different types of food (i.e., sugar and starch), increases in price resulted in roughly proportionate decreases in the consumption of both foods (Heyman et al. 1999). As a result, preference remained unchanged over a wide range of price and income levels.

That changes in alcohol and food consumption were uncorrelated over most price increases suggest the factors controlling consumption of each differed in one or more important ways. In the language of economics, the results suggest starch was not a “substitute” for the sweetened alcohol drink and vice versa, even though both substances were consumed in the same way. This observation can be tested by separately increasing the price of each substance. If they are substitutes, then consumption will shift to the one with the cheaper price. For example, econometric studies show that increases in the price of butter lead to an increase in margarine consumption and vice versa (e.g., Frank 1991). Thus, we arranged conditions so that the behavioral requirements for each substance increased one at a time. As suggested by the results shown in figure 1, increasing the price of alcohol did not result in increased consumption or preference for food, and conversely, increasing the price of food did not result in increased consumption or preference for alcohol. This is a surprising result given that both substances are ingested and provide calories. However, for humans, it is also the case that there are settings under which nonalcoholic drinks are poor substitutes for alcoholic ones. When a social drinker is at a bar or cocktail party, soft drinks are usually a distant second to wine and beer.

### Alcohol Regulated Preference: The Conservation Hypothesis

From the perspective that price increases are aversive, it is counter-intuitive that alcohol-reinforced responding increased. However, there is a principle that explains this apparent discrepancy. If the rats were motivated to maintain baseline levels of alcohol (but not starch), the results are quite sensible. Notice that the contingencies were structured so that it was possible to keep alcohol consumption levels constant if responding increased in direct proportion to the response requirement increments. Up to a threefold hike in response costs, this is exactly what happened at the alcohol lever. In other words, the quantitative features of the changes in behavior could be deduced from the assumption that the rats’ strongest motivation was to maintain a constant daily ration of alcohol.

If the conservation hypothesis is correct, then it should also be possible to arrange conditions that induce the rats to decrease their preference for alcohol. This was tested in a study that provided “free” servings of alcohol (Heyman 1993). As in the experiment associated with figures 1 and 2, lever presses earned either a sweetened alcohol drink or food. However, the chamber also held a dish filled with varying amounts of sweetened alcohol. The rats would first drink from the dish and then, after the dish was empty, start lever pressing for alcohol and food. The basic finding was that alcohol-reinforced responding declined, and the magnitude of the decrease was proportional to the amount of alcohol that was in the dish. Moreover, the total amount of alcohol consumed remained approximately constant at about 1.25 to 1.5 mL per session (which is about the same as the amounts earned in the experiment summarized by figures 1 and 2). In contrast, free alcohol did not lead to decreases in food consumption. These findings demonstrate that lever pressing was in the service of alcohol, decreasing or increasing as dictated by the experimental manipulations and the alcohol conservation principle.

The immediate goal of these experiments was to establish a model of human drinking. The criteria were (1) different patterns of food and alcohol consumption, (2) the persistence of alcohol consumption despite the availability of highly preferred competing activities, and (3) that increases in the cost of responding should have less of an effect on alcohol than on other normally effective reinforcers. It seems fair to say that these goals were met. In addition, in experiments like the ones just described, the rats developed tolerance to alcohol’s intoxicating effects as measured by tasks such as the ability to walk on a rod and to regain an upward posture when dropped backwards from a short height onto a pillow (Shegog 1991).

### Why Did Increases in Price Decrease Food But Not Alcohol Consumption?

Although the solutions were isocaloric, it is possible that alcohol’s food-related effects, such as its taste, motivated the rats. This line of inquiry is not likely to shed much light on alcohol abuse or dependence, but the issue needs to be addressed before pursuing ideas more likely to be relevant to human drinking. The experiments were based on the assumption that if demand for alcohol was based on taste, then it should be possible to show that taste is a sufficient condition for establishing inelastic demand.

In the first taste test, the two competing reinforcers were a sucrose solution and a sucrose plus bitter quinine solution (Heyman 1997). Initially, prices were the same for both solutions, and then each was increased in turn. If taste establishes a basis for inelastic demand, the rats should resist the lower price and stick with their initial, taste-based preferences. At first, the rats preferred the sucrose solution without quinine by a margin of about four to one. However, when the price of the quinine solution became relatively cheaper, the rats readily switched to it, despite their initial aversion. Conversely, when the price of the plain sucrose solution decreased, the rats readily increased their preference for it. Unlike the alcohol studies, there was no sign of inelastic demand. A study with two different foods, starch and sucrose (Heyman et al. 1999), showed the same pattern of results. Thus, flavor and macronutrient content did not establish the conditions for inelastic demand. This suggests that flavor and related factors do not explain inelastic demand in the alcohol studies.

### Pharmacologically Induced Selective Changes in Alcohol Consumption

**Figure 3:**
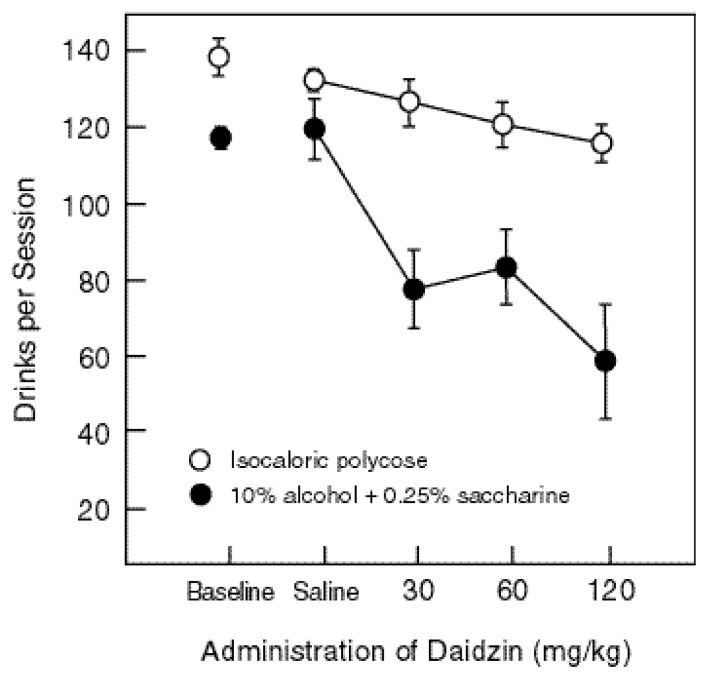
Administration of daidzin,* an isoflavone, decreased both alcohol and food consumption among study rats. The decreases in alcohol intake, however, were greater than the decreases in food consumption, and these differences increased in relation to dose. The data points show an average of seven rats. Each daidzin dose was administered three times, saline was administered five times, and baseline was defined as the session just preceding the saline session. *Daidzin is derived from a plant used by traditional Chinese herbalists to treat excessive drinking (Heyman et al. 1996).

Because alcohol consumption and food consumption are clearly distinguishable in the concurrent choice experiments just reviewed, the procedure provides a strategic baseline for identifying the biological mechanisms that mediate alcohol consumption. One study showed that Ro15-4513, a drug that acts at the same receptor (i.e., the docking) site on the cell membrane as the benzodiazepine tranquilizers Valium^®^ and Librium^®^, produced small but significant decreases in alcohol consumption at doses that did not decrease sucrose consumption (Petry 1995). Figure 3 below shows that daidzin, a naturally occurring isoflavone, produced greater changes. At each dose it reduced alcohol consumption, and the decreases in alcohol intake were always larger than the decreases in food intake (Heyman et al. 1996). Also, in this experiment blood alcohol was measured. The range was 50 to 304 mg/dl, and the average was 139 mg/dl. In other rat self-administration studies, blood alcohol levels are usually not greater than about 80 mg/dl (e.g., Schwarz-Stevens et al. 1991), and the criterion for drunk driving is usually between 80 and 100 mg/dl.

The results from this last study are of special interest because daidzin is a constituent of a traditional Chinese treatment for problem drinking. Chinese herbalists use the root of the kudzu plant to treat drinking problems (Keung and Vallee 1998*b*). Daidzin is derived from this root, and recently Dr. Keung and Dr. Vallee of Harvard Medical School found a correlation between daidzin’s ability to inhibit alcohol drinking in rodents and its ability to inhibit serotonin and dopamine metabolism in isolated liver mitochondria (Keung and Vallee 1998*a*). Thus, anecdotal reports of human behavior and in vitro laboratory findings are consistent with the animal model data. However, direct tests of daidzin’s influence on human alcohol consumption have yet to be undertaken.

## Implications: Does Economics Apply to Human Drinking?

Elementary economic ideas provided the conceptual framework for the experimental procedures described in this article. Might economics also provide a framework for understanding alcoholism?

According to the DSM, addiction, including alcoholism, is “compulsive” drug consumption. According to the dictionary (e.g., American Heritage 1992), a compulsion is an irresistible act that is either irrational or occurs regardless of its rationality. However, an implication of the analogy between inelastic demand and problem drinking is that alcohol dependence and alcohol abuse reflect preferences for alcohol not irresistible urges. For instance, inelastic demand is a function of the economic conditions, in particular the absence of substitutable goods or activities. Thus, if this viewpoint applies, it should be possible to arrange conditions that bring drinking in alcoholics to a halt. Several lines of evidence support this inference.

A series of experiments conducted in the 1970s showed that price increases would curb drinking in alcoholics (e.g., Bigelow and Liebson 1972; Cohen et al. 1971*b*). In one of the more realistic studies, alcoholics were given a priming dose of alcohol and a monetary reward if they drank no more than the initial drink (Cohen et al. 1971*a*). The subjects opted for the monetary reward, but as the concentration of the initial priming dose increased, the size of the monetary reward required for abstinence increased. That is, the alcoholics had a price, and the price depended on the pharmacological impact of the drink. Also note that the alcoholics were more sensitive to price increases than the rats in the laboratory studies. According to economic theory this means that in the human study there were more activities that could substitute for drinking than in the rat study. This seems a reasonable explanation, as the rats lived in an environment where there were only a few obvious reinforcing activities: eating chow, eating starch, drinking alcohol, and drinking water.

The human studies were conducted in isolated residential settings, and it is legitimate to point out that although these settings may have been richer than those in the rat studies, they were artificial. However, such criticisms miss the point that cost-benefit contingencies curbed drinking in alcoholics—even when the alcohol was accessible and even when some drinking had already occurred. On the basis of data such as these, Vuchinich and Tucker (1988) demonstrated that choice theory can describe alcoholic drinking patterns, and in a series of papers on relapse and abstinence, they extended the 1970s laboratory findings to natural settings (e.g., Tucker et al. 1995).

If economics and choice theory describe alcoholic drinking patterns, then social drinking and alcoholism differ in degree rather than kind. For example, both social and alcoholic drinking can be described in terms of the number of situations in which demand for alcohol is inelastic. For social drinkers, demand is somewhat inelastic in settings such as bars and cocktail parties, but not at home in the early morning or afternoon. In contrast, for many alcoholics, demand for alcohol is inelastic at bars and at home in the early morning. Put another way, social drinkers inhabit more settings in which alcohol has a substitute.

Finally, it should be pointed out that the economic analysis does not deny the importance of biological influences on drinking or the potential value of an effective pharmacological treatment for alcoholism. For instance, economic approaches to addiction imply that treatment should include practices that strengthen behaviors that will substitute for the drug (e.g., Higgins et al. 1994). This approach would be greatly facilitated by pharmacological agents that attenuated or blocked the immediate positive effects of the drug. This would indirectly increase the value of competing activities, and periods of sobriety would give such activities time to take root. Thus, economics provides principles for developing effective animal models of human drinking and a framework for fitting together the many biological and experiential factors that influence human drinking, including alcoholism.
